# The Effects of Gynecological Tumor Irradiation on the Immune System

**DOI:** 10.3390/cancers16162804

**Published:** 2024-08-09

**Authors:** Jesus Romero Fernandez, Sofia Cordoba Largo, Raquel Benlloch Rodriguez, Beatriz Gil Haro

**Affiliations:** Radiation Oncology Department, Hospital Universitario Puerta de Hierro, C. Joaquín Rodrigo 1, 28222 Majadahonda, Spain; sofia.cordoba@salud.madrid.org (S.C.L.); raquel.benlloch@salud.madrid.org (R.B.R.); bgilh@salud.madrid.org (B.G.H.)

**Keywords:** radiobiology, radiotherapy, radiation-induced immune effects, abscopal effect, gynecological cancer

## Abstract

**Simple Summary:**

Radiobiology has evolved from a mechanistic model based on DNA damage, and other response factors, into a more complex model including effects on the immune system and the tumor microenvironment (TME). Irradiation has an immunomodulatory effect that can manifest as increased anti-tumor immunity or immunosuppression. Irradiation promotes anti-tumor immunity through pro-inflammatory cytokines and endothelial damage, the recruitment of immune cells, and radiation-induced immunogenic cell death (ICD), characterized by the release of damage-associated molecular patterns (DAMPs) and tumor antigens. Irradiation activates both the innate and adaptive arms of the immune system. Irradiation also produces immunosuppression via the recruitment and activation of immune cells, with immunosuppressive effects. In this work, we discuss the mechanism involved in radiation-induced immune effects on which the combination of radiotherapy and immunotherapy for gynecological cancers is based.

**Abstract:**

Radiobiology has evolved from a mechanistic model based on DNA damage and response factors into a more complex model that includes effects on the immune system and the tumor microenvironment (TME). Irradiation has an immunomodulatory effect that can manifest as increased anti-tumor immunity or immunosuppression. Irradiation promotes an inflammatory microenvironment through the release of pro-inflammatory cytokines and endothelial damage, which recruit immune system cells to the irradiated area. Radiation-induced immunogenic cell death (ICD), characterized by the release of damage-associated molecular patterns (DAMPs) and tumor antigens, triggers an anti-tumor immune response of both innate and adaptive immunity. Anti-tumor immunity can manifest at a distance from the irradiated area, a phenomenon known as the abscopal effect (AE), which involves dendritic cells and CD8+ T cells. Irradiation also produces an immunosuppressive effect mediated by tumor-associated macrophages (TAMs) and regulatory T lymphocytes (Tregs), which counterbalances the immunostimulatory effect. In this work, we review the mechanisms involved in the radiation-induced immune response, which support the combined treatment of RT and immunotherapy, focusing, where possible, on gynecologic cancer.

## 1. Introduction

More than 60% of cancer patients receive radiotherapy during their disease, and up to 40% of cured patients have received this treatment [1]. Radiotherapy, whether combined with chemotherapy or not, is part of the standard treatment of various gynecological tumors [2,3,4,5]. Generally, two modalities of radiotherapy application are combined: external-beam radiation therapy (EBRT) and brachytherapy (BT), the latter consisting of the introduction of radioactive material into cavities or interstitially. The role of curative radiotherapy alone or combined with surgery and/or chemotherapy has been fully established in many other tumors for which it is a standard treatment; however, many aspects of its effects at the cellular and molecular levels are not fully understood.

Despite advances in surgical techniques and radiotherapy, some patients with gynecological tumors continue to experience disease recurrence. Depending on the stage, 30–70% of patients with locally advanced cervical cancer [6] and 20–80% of locally advanced endometrial adenocarcinomas will relapse [7], making these tumors a therapeutic challenge. Recent advances in the diagnosis and individualized treatment of gynecological tumors include sentinel node biopsy (SNB) [8], which reduces the morbidity of lymphadenectomy, and the molecular classification of endometrial cancer [9], which allows the selection of personalized treatment based on the gene mutation profiles of tumors [10]. The major recent advance in the treatment of gynecological tumors has been the combination of immunotherapy with chemotherapy and/or radiotherapy, whose biological basis will be discussed in this review.

Radiobiology has evolved in recent decades from a mechanistic model into a model based on more precise knowledge of the cellular and molecular mechanisms triggered by irradiation. This knowledge allows the design of therapeutic strategies that improve radiotherapy results.

The enormous development of immunotherapy in the last decade has greatly increased interest in understanding the immune system’s anti-cancer cell mechanisms. Preclinical data and clinical investigations supporting the benefit of combining immunotherapy with radiotherapy in gynecological tumors have sparked interest in searching for therapeutic strategies to enhance anti-tumor immunity.

In this paper, we explore the radiobiology of gynecological tumors from classical radiation response mechanisms to new insights into the immune response triggered by irradiation.

## 2. Classical Radiobiology: The “5Rs” Model

The classical theory of radiobiology states that the effects of radiation on cells occur via the interaction of the irradiation beam with the DNA double-strand (direct effects) or via the formation of free radicals or reactive oxidative species (ROS) (indirect effects). The cellular response depends on DNA damage, which can result in cell death via apoptosis, necrosis, mitotic catastrophe, or senescence if not properly repaired [11]. It soon became apparent that other cellular mechanisms play a role in the response of tissues and tumors to irradiation. Aiming to create a short list of the mechanisms that determine the response of biological tissues to irradiation, Steel et al. [12] coined the so-called five Rs of radiobiology in 1989: the repair of sublethal DNA damage; redistribution with cell accumulation in the G2M phase of the cell cycle; reoxygenation, consisting of increased O_2_ pressure in hypoxic tumor cells as treatment progresses; the regeneration of both tumor (tumor repopulation) and healthy tissues produced by an increased proliferation rate; and radiosensitivity intrinsic to each cell line.

Fractionated radiotherapy is based on the greater repair capacity of normal tissues compared with tumors. Healthy tissues take advantage of their greater repair capacity between fractions, while tumors repair very little. Dose fractionation to preserve healthy tissues gave rise to the concept of the therapeutic index, which has two components: the tumor component and the healthy tissue component [13]. The therapeutic index is established by comparing the sigmoidal dose–response curve for tumor control with that for toxicity.

**Figure 1:**
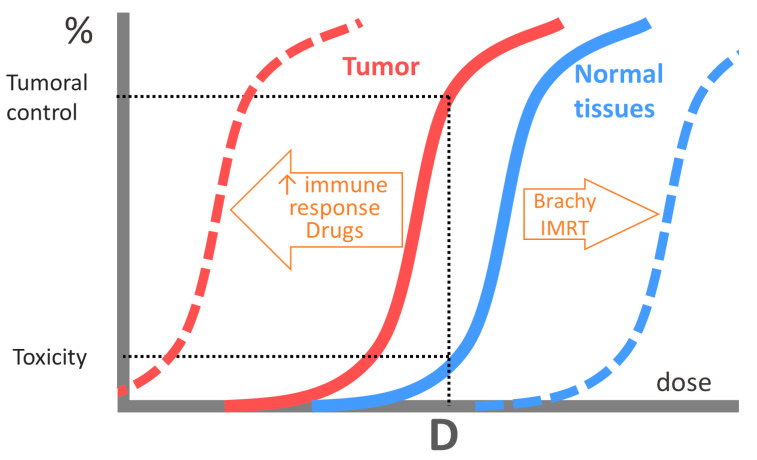
Therapeutic index: the therapeutic index is established by comparing the sigmoidal dose–response curve for tumor control (red) with that for toxicity (blue). The dashed lines represent the modification of the dose-response curves due to the antitumor immune response (red) or to techniques that spare healthy tissues such as IMRT or brachytherapy (blue).

For a treatment to be considered favorable, the two curves should be as far apart as possible. This allows the most effective dose for the tumor with a low level of complications. The therapeutic index can be improved by administering drugs that enhance tumor radiosensitivity; using more effective radiotherapy schedules; or using strategies that decrease the toxicity of healthy tissues, such as radioprotectors or highly conformal irradiation techniques. Brachytherapy is characterized by a heterogeneous distribution and a high dose gradient, which allows the sparing of healthy tissues and improves the therapeutic index (Figure 1).

Due to their cytotoxic potential, double-strand DNA breaks (DSBs) are the most relevant to the fate of the irradiated cell. Additionally, irradiation can also produce base loss or single-strand DNA breaks that are repairable via the base excision repair (BER) pathway. The two main repair pathways for radiation-induced DSBs are the nonhomologous end-joining (NHEJ) error-prone pathway used by cells in the G1 phase of the cell cycle and the homologous recombinational (HR) error-free pathway in dividing cells [14]. Irradiation damage is mainly repaired via the NHEJ pathway, which is a coordinated mechanism activated after DSBs alongside the activation of cell cycle checkpoints [15]. Takada et al. [16] have shown that a low expression of Ku86 and XRCC4, proteins involved in the NHEJ pathway, correlates with a better rate of complete remission in patients with cervical cancer treated with preoperative radiotherapy. The same group confirmed in a later work that low XRCC4 expression was associated with better prognosis in cervical cancer patients treated with chemoradiotherapy (CRT) [17]. Low expression levels of Ku-70, a key protein in the NHEJ repair pathway, were associated with improved disease-free survival in endometrial cancer patients treated with radiotherapy [18]. DNA damage can be repaired, resulting in cell survival; otherwise, in the case of defective repair, it results in mutations that can lead to cell death (lethal mutation) or non-lethal mutation that can lead to carcinogenesis (somatic cells) or malformations (fetal tissue).

### 2.1. Linear–Quadratic Model

The linear–quadratic model (LQM), which was developed based on experience in cell culture irradiation, was created to provide a mechanistic explanation for the biological effects of cell death and the repair of sublethal damage [19]. The model assumes a correlation between the irradiation dose and the resulting DNA damage.

The LQM formula describes the shape of the cell survival curve, which is determined by two parameters: α, representing a non-repairable lesion produced by a single irradiation event (the linear component), and β, representing several repairable lesions produced by independent events (the quadratic component).

**Figure 2:**
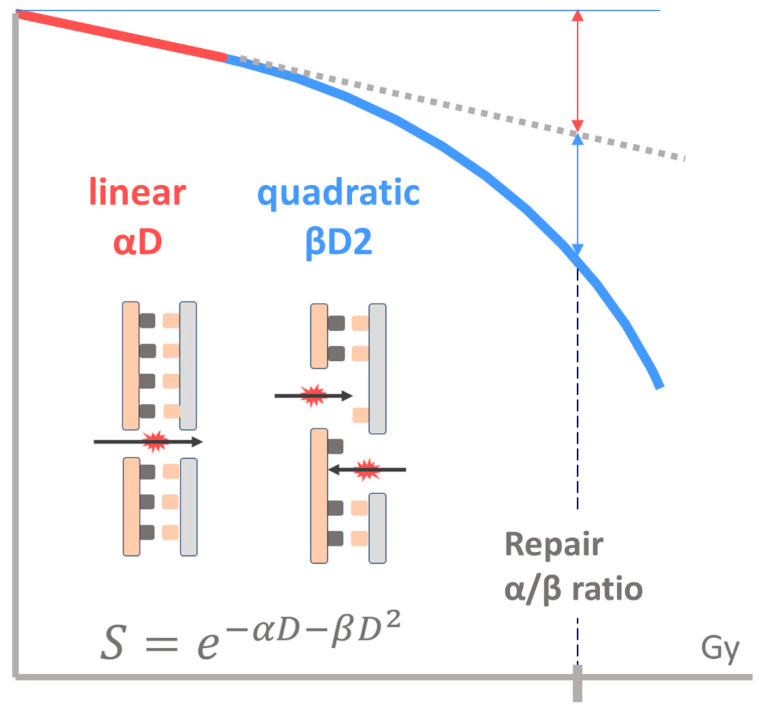
Linear–quadratic model. α parameter (red) represents a non-repairable lesion produced by a single irradiation event (linear component), and β parameter (blue) represents several repairable lesions produced by independent events (quadratic component).

The wider the shoulder of the survival curve, the greater the repair capacity of the cell or tissue, and the lower the so-called α/β ratio. The LQM’s consideration of the higher repair capacity (lower α/β ratio) of late-response tissues compared with acute-response tissues and tumors is key to its clinical applicability. The higher repair capacity of healthy tissue versus tumors is the basis for fractionation in radiotherapy (Figure 2).

The LQM allows the calculation of the biologically effective dose (*BED*) with the following formula:

$$BED=D\times 1+\frac{df}{\alpha /\beta }$$

where *D* represents the total dose; *df* represents the dose per fraction; and *α*/*β* represents the alpha/beta ratio.

Using the BED allows for the calculation of iso-effective doses to compare treatments with different fractionation schemes. The clinical use of the LQM has been widely validated in recent decades and is currently used to compare schemes with different fractionations or for dose adjustment in clinical practice. It is also useful for maintaining healthy tissue doses at safe levels when altered fractionation schedules are designed in clinical trials [20]. Radiobiological dose prescription has now been adopted in gynecological cancer brachytherapy planning [21]. The radiobiological dose considers the reparative capacity of tissues and tumors and, therefore, better predicts treatment outcomes [22].

In practice, the dose is prescribed as the equivalent to conventional irradiation administered at 2 Gy/fraction (*EQD*2):

$$EQD2=\frac{BED}{1+\frac{2}{\alpha /\beta }}$$

### 2.2. Regeneration 

Repopulation, consisting of accelerated cell division, is a mechanism developed by healthy tissues and tumors to compensate for radiation-induced death. While this phenomenon protects healthy tissues, in tumors, it is called accelerated tumor repopulation and is a recognized cause of radiotherapy failure. Accelerated tumor repopulation can occur due to interruptions or by lengthening the time between EBRT and brachytherapy or between fractions of brachytherapy. Within gynecological tumors, tumor repopulation is more important in cervical and vaginal tumors, while it has less impact on endometrial cancer. It has long been known that lengthening the overall treatment time (OTT) is associated with worse cervical cancer control [23]. The EMBRACE study estimated that an additional 5 Gy is needed to compensate for a 1-week lengthening of the OTT [24]. In vaginal cancer, lengthening the OTT beyond 9 weeks significantly worsens the pelvic control rate, decreasing from 97% to 54% (*p* = 0.0003) [25]. To account for accelerated tumor repopulation, the LQM incorporates a repopulation factor that is multiplied by the OTT minus the interval to the start of repopulation:

$$BED=D\times 1+\frac{df}{\alpha /\beta }-\left(\frac{ln2}{\alpha Tpot}\left(OTT-Tk\right)\right)$$

where *D* represents the total dose; *df* represents the dose per fraction; *α*/*β* represents the alpha/beta ratio; α represents the alpha parameter; *Tpot* represents the potential doubling time; and *Tk* represents the onset of the repopulation interval.

Radiobiological parameters vary widely depending on the type of study [26], although using generic values for the *α*/*β* ratio (3 for healthy tissues and 10 for tumors) is adequate for clinical use. Since the values of the parameters α and Tpot are highly variable [26], a repopulation factor (*ln*2/*αTpot*) value of 0.69 Gy/D for cervical cancer is acceptable [22]. A *Tk* value of 19 days (95% CI: 11–22) has been estimated by analyzing tumor volumetric regression data from 80 cervical cancer patients who received external beam radiotherapy (EBRT) and LDR brachytherapy [27].

Although most patients are currently treated with high-dose-rate brachytherapy (HDR-BT), in which the dose rate effect is negligible, the formula has been modified to account for the time factor to apply the LQM to low-dose-rate brachytherapy (LDR-BT) treatments [28,29].

### 2.3. Redistribution

The cell uses cell cycle checkpoints to try to repair DNA damage and, in case of irreparable damage, to initiate the cell death process. There are two cell cycle checkpoints: one at the G1/S level (controlled by the tumor suppressor gene p53) and the other at G2/M (regulated by the Cdk1-cyclin B complex) [30]. Since p53 mutation is frequent in cervical cancer, cell cycle arrest is more likely to occur in the G2/M phase of the cell cycle after each radiation dose.

Redistribution refers to this accumulation of tumor cells in the G2M phase of the cell cycle produced by irradiation, which has been confirmed in preclinical studies [31]. Early studies showed that G2M is the most radiosensitive phase of the cell cycle [32]. The drug combination that produces an arrest in the G2M phase of the cell cycle with radiotherapy exploits this phenomenon [33]. Theoretically, redistribution would favor fractionated treatment or LDR-BT over hypofractionated treatments or HDT-BT, as subsequent doses or low dose rates would allow for a greater number of more radiosensitive G2M-phase cells. However, clinical evidence has demonstrated the equality or superiority of stereotactic body radiation therapy (SBRT) and HDR-BT over conventional treatments, highlighting the need to look for other factors on which the response to radiotherapy depends.

Technical improvements since the 2000s have allowed the introduction of SBRT, characterized by using high doses per fraction administered in a few sessions. The good clinical results of SBRT challenged the concepts of radiobiology that suggested the benefit of fractionated radiotherapy to protect healthy tissues. Due to the use of high fraction doses, some authors have questioned the applicability of the LQM to hypofractionated SBRT schedules [34]. Nevertheless, the LQM has been successfully used in different studies for conversion to a single dose and comparisons between different schedules. Some authors have advocated for the LQM’s usefulness in fraction dose treatments of up to 18 Gy [35].

## 3. Radiation-Induced Immune Response

Advances in molecular research over the last two decades have provided insight into the complex relationships established between the tumor cell and the surrounding TME, which play a critical role in the tumor response and toxicity produced by irradiation [36]. The immune response, vascular damage, and the influence of hypoxia and radioresistance signals generated by the TME are some closely interconnected responses to irradiation that form a complex network.

Following tradition, the sixth R of radiobiology has been added to the existing list [37]: the reactivation of the anti-tumor immune response. This term refers to the ability of radiation to exert an immunomodulatory effect by initiating and maintaining an anti-tumor immune response, as well as triggering immunosuppression. This is a promising mechanism to be exploited in the clinical setting by combining radiotherapy with immune checkpoint inhibitors [38].

In [39], a seventh term was added to the list of Rs in radiobiology: the reinforcement of the TME. This term refers to how TME components support cancer cells against radiation therapy’s effects. The cellular components of the TME associated with radiation resistance are cancer-associated fibroblasts (CAFs), myeloid-derived suppressor cells (MDSCs), TAMs, tumor-associated neutrophils (TANs), and regulatory T cells (Tregs), in addition to the noncellular extracellular matrix (ECM). More research must be conducted in this area until we can develop strategies to reverse the radioresistance of tumor cells due to the influence of the TME [36].

In addition to the classical response mechanisms discussed above, abundant evidence indicates that the activation of the immune system triggered by irradiation contributes to cancer cell death. Irradiation can stimulate both the innate and adaptive immune system arms responsible for host protection against external threats or cancer. Irradiation acts at almost every level in the cancer immunity cycle. Its actions include promoting the release and presentation of tumor antigens, promoting lymphocyte priming and activation, increasing the number of tumor-infiltrating lymphocytes, and facilitating the recognition of tumor cells by T cells [40]. The anti-tumor effect produced by the activation of the immune system via radiotherapy is counterbalanced by the immunosuppression effects that can lead to an equilibrium situation or, in some cases, the evasion of the immune attack on the tumor.

**Figure 3:**
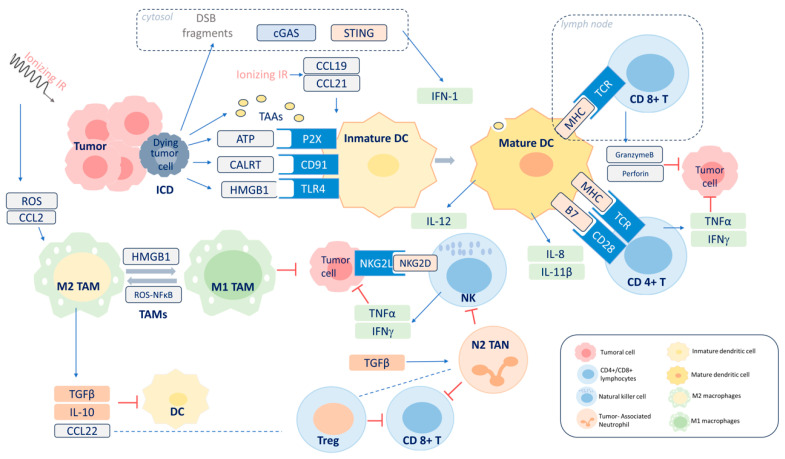
Schematic representation of the complex network of the immune response pathways triggered by irradiation. DSB: double-strand DNA break; cGAS: cyclic GMP-AMP synthase; STING: stimulator of interferon genes; IFN: interferon; CCL2, CCL19, CCL21, and CCL22: chemokines; TAAs: tumor antigens; ICD: immunogenic cell death; ATP: adenosine triphosphate; CALRT: calreticulin; HMGB1: high-mobility group protein B1; P2X, CD91, and TRL4: pattern recognition receptors; DC: dendritic cell; MHC: major histocompatibility complex; TCR: T-cell receptor; TNFα: tumor necrosis factor-alpha; ROS: reactive oxygen species; IL: interleukin; TGFβ: transforming growth factor-beta; NFκB: nuclear factor kappa B; TAM: tumor-associated macrophage; TAN: tumor-associated neutrophil; Treg: regulatory T lymphocyte.

The most widely accepted model considers that irradiation triggers an inflammatory microenvironment that favors ICD, characterized by the release of tumor antigens and damage-associated molecular patterns (DAMPs) that trigger the release of pro-inflammatory cytokines. This environment attracts myeloid and lymphoid cells that become effector cells with anti-tumor activity. Adaptive immunity is also generated following the activation of T helper (Th) and T effector (Teff) cells by dendritic cells (DCs). The immunostimulatory effects are counterbalanced by an immunosuppressive effect that is also triggered by irradiation executed by certain cytokines and regulatory T lymphocytes (Tregs) (Figure 3).

### 3.1. Inflammatory Microenvironment

Local irradiation triggers the production of pro-inflammatory cytokines, mainly IL-1β and TNF [41], and the recruitment of immune cells, triggered by endothelial expression of intercellular adhesion molecule 1 (ICAM1) and vascular cell adhesion molecule 1 (VCAM1) [42]. The action of IFNγ produced by infiltrating T lymphocytes increases the expression of endothelial adhesion molecules [43]. IFNγ also induces the production of chemokines, which exert their chemotaxis action by recruiting T lymphocytes to the TME [44]. These changes are intracellularly controlled by ROS and nuclear factor κ B (NFκB) [42].

In addition to its direct cytotoxic potential, DNA damage produced by irradiation can induce the release of proinflammatory cytokines. Cytosolic double-stranded DNA fragments become a danger signal due to their ability to bind to cyclic GMP-AMP synthase (cGAS) and activate the adaptor protein stimulator of interferon genes (STING) [45]. The activation of the cGAS-STING pathway leads to the production of type I interferons and other proinflammatory cytokines [46] that favor DC activation and the cross-presentation of tumor-derived antigens to T cells [47].

### 3.2. ICD

Radiation can induce ICD, a type of cell death that triggers immune responses to specific antigens on the tumor cell [48,49]. The inflammatory microenvironment and cell death produce an immune response from the release of tumor antigens and DAMPs [50,51] and their corresponding pattern recognition receptors (PRRs). The expression of calreticulin in the cell membrane [52], the active secretion of ATP, and the cytoplasmic translocation and extracellular relay of high-mobility group protein B1 (HMGB1) are among the DAMPs induced by irradiation [53]. PRRs include the family of toll-like receptors (TLRs), located in the membranes of immune system cells such as macrophages, DCs, and natural killer cells. The activation of TLRs triggers transcription factors such as NFκB which regulate the production of cytokines and interferons with various functions [54]. The release of HMGB1 activates the TLR4 receptor in DCs, which favors their maturation by increasing their endocytic activity and cross-presentation by which exogenous tumor-derived antigens are presented to T cells on major histocompatibility complex (MHC) class I molecules [55]. The release of IL-1β and IL-8 by DCs, stimulated by ATP produced after irradiation, favors cross-presentation [52]. The production of DAMPs via irradiation is dose-dependent and stimulates both the recruitment and phagocytic activity of tumor antigens by DCs [56]. Additionally, ionizing radiation induces NKG2D ligands and, consequently, might lead to increased natural killer (NK) cell-mediated cytotoxicity [57]. A study in cervical cancer patients comparing the effects of HDR-BT with pulsed-dose brachytherapy (PDR-BT) on the immune cell population in peripheral blood found that HDR-BT produced significantly higher values of CD56dimCD16 + NK, which indicated a greater cytotoxic capacity than PDR-BT [58].

### 3.3. Adaptive Immunity

Adaptive immunity is the function of B and T lymphocytes, which generate antigen-specific responses and immunologic memory. These responses are generated by the interactions between antigen-specific receptors on B cells and T cells. Concerning T lymphocytes, the T-cell receptors (TCRs) CD4 and CD8 recognize a specific MHC–peptide complex presented by an antigen-presenting cell (APC). T-cell activation occurs upon reaching a certain threshold of interactions between the TCR and the MHC–peptide complex. Co-stimulatory signals are also required, such as the binding of the CD80/86 ligand on DCs to the CD28 protein on T cells. The upregulation of the CTL4 receptor on T cells has an inhibitory effect that is critical for T-cell homeostasis and self-tolerance by capturing and removing CD80/86 from APCs [59]. CD8+ cytotoxic T cells mediate direct tumor cell death by releasing granzyme B and perforin, whereas CD4+ helper cells secrete IFNγ, IL17, and IL2 [60]. An in vitro study found a radiation-induced increased expression of the MHC class via elevated IFNβ signaling [61]. Irradiation stimulates tumor cells to release chemokines (CXCL16 and CXCL10) that activate T cells and increase their recruitment into the tumor [62]. Adaptive immunity’s ability to recognize and kill tumor cells has led to the consideration of local irradiation as an in situ anti-tumor vaccination.

Several studies have observed that infiltration by CD8+ T cells correlates with increased tumor control in cervical cancer [63,64]. Someya et al. analyzed TME immunity in cervical cancer patients treated with CRT and found that inflamed-type tumors (>30 CD8+ T cells/HPF in tumors) and excluded-type tumors (>30 CD8+ T cells/HPF in stroma but not in tumors) were associated with a better 5-year disease-specific survival (DSS) than cold-type tumors with poor CD8+ T cell infiltration (*p* < 0.001; 5-year DSS; 60.3% vs. 72.3% vs. 0%, respectively) [17]. A study using immunohistochemistry of tumor biopsies from patients with cervical carcinoma showed that weak HLA-A-MICA expression combined with a low CD8+ T cells/Treg ratio was associated with worse survival [65].

### 3.4. AE

Some evidence indicates a systemic anti-tumor effect of local irradiation, known as AE. AE is a rare manifestation of immune system activation caused by local irradiation [66], characterized by the regression of tumors or metastases distant from the irradiation field of the primary tumor. A systematic review identified only 46 cases of AE between 1969 and 2024 [67]. There is only one published case of cervical cancer of AE consisting of the clinical remission of non-irradiated retroperitoneal disease after pelvic radiotherapy [68]. The occurrence of true AE, i.e., without immunotherapy treatment, has rarely been described, and the immune system’s involvement in this phenomenon has been suspected from the beginning. In the last decade, much research has been carried out on the biological basis of EA and the factors that determine its occurrence to determine its potential clinical impact.

In vivo studies have demonstrated the involvement of DCs in AE [69,70]. Meanwhile, irradiation has demonstrated an ability to activate the HMGB1/TNFα signaling pathway promoting the polarization of macrophages into the proinflammatory M1 type, which could act at distant sites [71]. An in vivo murine model demonstrated CD8+ T cells’ participation and the negative effect of TGFβ on the appearance of AE [72]. ICAM1 upregulation has been observed in non-irradiated tumors away from the irradiation site, leading to the activation and infiltration of CD8+ T cells in non-irradiated areas [73]. Given the role of tumor-specific T cells, it has been found that AE is enhanced when irradiation is combined with a CTL-4 blockade in both animal [74] and human [75] models.

Ji et al. [76] observed that immunosuppressive cells play a negative role in AE. The study showed that combining anti-CD25 and radiotherapy increased CD8+ T cells and reduced Treg cell infiltration, enhancing the ability to kill at a distance [76]. Other factors influencing AE have recently been reviewed in [77].

### 3.5. Radiation-Induced Immunosuppression

The immunomodulatory effects of ionizing radiation are immunostimulatory and can promote immunosuppression in certain conditions. Signals triggered by irradiation produce anti-inflammatory cytokine signaling (IL-2 and TGFβ); the recruitment of TAMs with immune suppressive activity; myeloid-derived suppressor cells (MDSCs); and Tregs [78]. Treg cells, a subgroup of CD4+ T lymphocytes, secrete the cytokines TGFβ and IL-10 with effector T-cell suppressive activity and stimulate the suppressive function of MDSCs [79,80]. The increased radiosensitivity of effector T cells relative to regulatory T cells increases the depletion of anti-tumor lymphocytes [81]. Qinfeng et al. analyzed the various immune populations in biopsies from cervical cancer patients taken at different times during radiotherapy and found a decrease in CD8+ and CD4+ T cells along with an increase in FOX3P+ T cells, suggesting that Tregs are more radioresistant than Teff cells [82]. Consistent with this observation, elective lymph node irradiation in a murine model was shown to worsen the Teff/Treg ratio and tumor control [83]. Peripheral blood lymphocyte depletion in glioma patients treated with radiotherapy appears to depend on the volume and dose of radiotherapy [84].

There is current interest in studying the effects of programmed death ligand 1 (PD-L1) on treatment outcomes in gynecological cancers. Preclinical studies have shown that human papillomavirus (HPV) infection in cervical cancer can lead to immune evasion via the activation of the PD-L1/PD-1 signaling pathway. Liu et al. [85] demonstrated that the overexpression of the HPV16E7 oncoprotein in epithelial carcinoma of PC3 cells increased the expression level of the PD-L1 protein and inhibited peripheral blood mononuclear cell (PBMC) proliferation and cytotoxic T-lymphocyte (CTL) activity. Iijima et al. showed that PD-L1 expression was upregulated by a C-ion beam in a dose-dependent manner in HeLa and SiHa cells [86]. Moreover, PD-L1-expressing tumor cells significantly increased after CRT in cervical cancer patients [87].

A study in cervical cancer patients who received radiotherapy showed that successful treatment was associated with lower PD-L1 and CD163 levels, a characteristic of alternatively activated macrophage type M2, compared with patients who did not respond well [88]. That study also found significant differences in 2-year progression-free survival in a multivariate analysis [88]. Considering these data along with the high PD-L1 expression exhibited in 34% of cervical tumors [89], further investigation into combining radiotherapy with immune checkpoint inhibitors is warranted.

## 4. Clinical Perspectives

Immunotherapy, administered as monotherapy, fails in more than 70% of patients due to tumor cells’ ability to evade the immune system. Radiotherapy can increase the number and variety of tumor antigens and start both the innate and adaptive immunity machinery. Thus, combining radiotherapy and immunotherapy could improve disease control and overcome immune system evasion.

HPV-associated cervical cancer presents a state of immunosuppression due to PD-1/PD-L1 upregulation, making it a good candidate for the use of immune checkpoint inhibitors. Following the KEYNOTE-826 study, pembrolizumab was approved for use in metastatic cervical cancer patients [90].

Ipilimumab, a CTL-4 pathway inhibitor, has shown efficacy when combined with radiotherapy in preclinical [91] and clinical studies [92].

In the phase I trial GOG-9929, patients with node-positive cervical cancer treated with CRT followed by ipilimumab achieved a 1-year OS of 90% [93]. An increase in PD-1 expression on CD4- and CD8-positive T cells was observed after CRT, which was suppressed after ipilimumab administration [94].

Preclinical studies have demonstrated the efficacy of both anti-PD-1 antibodies [95] and PD-L1 blockade [96] combined with radiotherapy. A recent phase III trial comparing pembrolizumab or a placebo with CRT followed by pembrolizumab or a placebo for newly diagnosed, high-risk locally advanced cervical cancers demonstrated significantly improved progression-free survival with this combination [97].

The combination of immunotherapy and radiotherapy is a very active area of investigative research that may change the approach to gynecological tumors in the coming years. Different drugs and therapeutic schemes are being tested in different gynecological cancer sites [38].

## 5. Conclusions

Radiobiology, the basic science of radiotherapy, was initially based on radiation-induced DNA damage and the ability of tumors and healthy tissues to repair this damage. DNA damage repair, along with other radiotherapy response factors, was grouped into a list known as the 5Rs of radiotherapy. Redistribution, regeneration, reoxygenation, and radiosensitivity were added to repair. The MLQ is a mechanistic model based on the ability to repair DNA damage that remains valid and is well suited to the clinical setting. Modern radiobiology has evolved into a more complex model that considers the immunomodulatory effect of irradiation and the effects of SMT on the tumor response in addition to DNA damage. Irradiation causes the release of pro-inflammatory cytokines that attract immune cells that exert an immunostimulatory action. ICD produced by irradiation triggers the activation of both innate and adaptive immunity that exerts anti-tumor effects. Irradiation also produces a state of immunosuppression exerted by cells with an immunosuppressive capacity, such as TAMs, CAFs, and Tregs.

Knowledge of these factors has contributed to the establishment of a combination of immune checkpoint inhibitors with radiotherapy as a standard treatment in some gynecological cancers. Ongoing clinical trials will define the role that this strategy will play in the management of gynecological tumors.
